# Mid-Regional Pro-Adrenomedullin (MR-proADM) as a Biomarker for Sepsis and Septic Shock: Narrative Review

**DOI:** 10.3390/healthcare6030110

**Published:** 2018-09-03

**Authors:** Uğur Önal, Francisco Valenzuela-Sánchez, Kalwaje Eshwara Vandana, Jordi Rello

**Affiliations:** 1 Department of Infectious Diseases and Clinical Microbiology, Faculty of Medicine, Ege University, 35100 Izmir, Turkey; ugur.onal@ege.edu.tr; 2 Critical Care Medicine Department, University Hospital of Jerez, 11407 Jerez de la Frontera, Spain; pacovaes@yahoo.es; 3 Department of Microbiology, Kasturba Medical College, Manipal Academy of Higher Education, Manipal 576104, India; 4 Vall d’Hebron Institut of Research, CIBERES, 08035 Barcelona, Spain; jrello@crips.es

**Keywords:** mid-regional pro-adrenomedullin (MR-proADM), sepsis, septic shock

## Abstract

Early identification and diagnosis of sepsis and septic shock is vitally important; despite appropriate management, mortality and morbidity rates remain high. For this reason, many biomarkers and screening systems have been investigated in accordance with the precision medicine concept. A narrative review was conducted to assess the role of mid-regional pro-adrenomedullin (MR-proADM) as a biomarker for sepsis and septic shock. Relevant studies were collected via an electronic PubMed, Web of Science, and The Cochrane Library search. The review focused on both diagnosis and prognosis in patients with sepsis and septic shock and specifically in subpopulations of patients with sepsis and septic shock with burns or malignant tumors. No exclusion criteria regarding age, sex, intensive care unit admission, follow-up duration, or co morbidities were used so as to maximize sensitivity and due to lack of randomized controlled trials, opinion paper and reviews were also included in this review. A total of 22 studies, one opinion paper, and one review paper were investigated. MR-proADM levels were found to be useful in assessing patients’ initial evolution and become even more useful during follow-up with increased area under curve values in the mortality prognosis by exceeding values of 0.8 in the data shown in several studies. These results also improve along with other biomarkers or severity scores and especially correlate with the organ failure degree. The results of this study indicate that MR-proADM is a good biomarker for the diagnosis and prognosis of sepsis and septic shock patients as well as for organ failure. Although several publications have discussed its role as a biomarker for pneumonia, its value as a biomarker for sepsis and septic shock should now be assessed in randomized controlled trials and more collaborative prospective studies with larger patient samples.

## 1. Introduction

Adrenomedullin (ADM) plays an important role in the inflammation process and in progression from sepsis to septic shock. Secreted from various organs and tissues, it is mainly produced by vascular endothelial cells and plays a part in vasodilator, positive inotropic, diuretic, natriuretic, and bronchodilator functions [1,2]. Because of the rapid clearance of circulating ADM from the circulation and its binding protein (complement factor H), it is difficult to detect plasma ADM levels by standard immunoassay. The mid-regional fragment of pro-adrenomedullin (MR-proADM) is more stable and demonstrates the levels of the active ADM peptide [3].

Sepsis and septic shock remain a major healthcare problem worldwide, with high mortality rates. Early identification is vital for reducing these rates [4]. For this reason, many biomarkers and screening systems have been investigated in accordance with the precision medicine concept [5].

Despite the fact that the sepsis and septic shock definitions were updated and severe sepsis with systemic inflammatory response syndrome criteria have been passed out of use according to the third international consensus (Sepsis-3), sepsis is defined as organ dysfunction caused by dysregulated response of a host to an infection and the sequential organ failure assessment (SOFA) score scale has been approved for use in defining organ dysfunction. However, this scale has some limitations because several parameters need to be assessed and cut off points were established for intensive care unit patients [6]. Although there is no golden standard biomarker for sepsis and septic shock patients in terms of diagnosis and prognosis at present, MR-proADM seems to be a good alternative for SOFA score for the assessment of organ failure as well as for predicting mortality of septic patients.

In this paper, we review the role of MR-proADM as a biomarker for sepsis and septic shock. Our hypothesis was that MR-proADM may be a prognostic marker of severity in septic patients and has an important role to play in the initial evaluation and the clinical management of this condition.

## 2. Materials and Methods 

We conducted a literature review to investigate the role of MR-proADM in sepsis and septic shock, applying the methodology described below.

### 2.1. Information Sources

An electronic PubMed, Web of Science, and The Cochrane Library search was performed.

### 2.2. Search

The search was performed using the following strategy and key words: mid-regional pro-adrenomedullin [Title/Abstract] OR MR-proADM [Title/Abstract] AND septic shock [Title/Abstract] OR mid-regional pro-adrenomedullin [Title/Abstract] OR MR-proADM [Title/Abstract] AND sepsis [Title/Abstract].

### 2.3. Study Selection

Although new definitions of sepsis and septic shock are available [6], in the interests of comprehensiveness we also included the clinical criteria or confirmed diagnosis for sepsis, severe sepsis, and septic shock [7]. The three target conditions of interest for this review are defined as sepsis, severe sepsis, or septic shock. No exclusion criteria regarding age, sex, intensive care unit admission, follow-up duration, or co morbidities were imposed.

### 2.4. Data Items

We extracted data onto a study-specific pro forma, which included the following list of clinical questions under the PICO framework (Population-Intervention-Comparison-Outcome) format (Table 1). MR-proADM was identified as a diagnostic and prognostic marker, stratifying the mortality risk in septic patients. The measure of the index test as MR-proADM was performed in blood samples. The thresholds used in this review were those generated and presented within each study included, as there is currently no accepted standard; therefore, it was not possible to specify a threshold value beforehand.

### 2.5. Risk of Bias in Individual Studies

Studies were initially selected by the main author (UO), who performed title and abstract screening. We then obtained the full text of each potentially eligible study.

### 2.6. Summary Measures

Mean and median levels, odd ratios, and *p* values were stated for each included study, but a meta-analysis could not be performed due to the heterogeneity of the results.

### 2.7. Synthesis of Results

Analysis of all outcomes was performed, including the authors, year of publication, country, study design, total number and classification of patients, and the comparison of MR-proADM with other biomarkers or scoring systems. The results are also sorted by number of patients respectively.

### 2.8. Additional Analyses

The reference lists of all relevant studies were also checked.

### 2.9. Study Selection and Characteristics

A total of 22 studies, one opinion paper, and one review paper were investigated (Table 2).

### 2.10. Risk of Bias within Studies

Two articles were excluded due to the high risk of bias or concerns regarding applicability (using the QUADAS-2 tool) [8]. Additional screening identified one review and one opinion paper with no meta-analyses or randomized controlled trials.

As the study was designed as a narrative literature review, no ethical committee permission was needed.

## 3. Results

### 3.1. Usefulness of MR-ProADM as Biomarker for Diagnosis and Prognosis in Sepsis

Several biomarkers have been investigated for early diagnosis of sepsis [9,10]. In the literature, MR-proADM has mainly been associated with pulmonary infections [11,12,13,14,15,16].

A multinational, prospective, observational cohort study named TRIAGE included a total number of 7132 patients with medical emergencies shows that for the prediction of death, ProADM significantly improved regression models, also improved clinical models for prediction of intensive care unit admission and high initial treatment urgency [17].

Enguix-Armada et al. analyzed 388 patients to determine whether the combination of C-reactive protein (CRP), procalcitonin (PCT), presepsin, or SCD14-ST and MR-proADM measured in the first 24 h after intensive care unit (ICU) admission improved diagnostic and prognostic management in both severe sepsis and septic shock. They found that MR-proADM showed a better prognostic value (*p* < 0.001) particularly in cases of septic shock (*p* < 0.01), increasing in line with the APACHE-II score [18].

In the study by Andaluz-Ojeda et al. 326 patients with severe sepsis (21.7%) or septic shock (79.3%) were assessed in order to compare biomarkers (CRP, PCT, lactate, and MR-proADM) for septic patients with different degrees of organ failure (via SOFA scores). MR proADM was the only biomarker to predict mortality in all severity groups (SOFA ≤ 6, SOFA = 7–12, and SOFA ≥ 13) with AUROC (area under receiver operator characteristic) (95% CI (confidence interval) of 0.75 (0.61–0.88), 0.74 (0.66–0.83), and 0.73 (0.59–0.86), respectively (*p* < 0.05); the best AUROC was for mortality prediction at 28 days in the analysis over the entire cohort (AUROC (95% CI) 0.79 (0.74–0.84)) (*p* < 0.001) [19].

Recently, Charles et al. published a prospective cohort study of 173 septic patients admitted to ICU in order to evaluate the prognostic values of PCT, (MR-proADM, copeptin, and CT-pro-endothelin 1 (CT-ProET 1) concentrations. The best AUROC values on day one were obtained with MR-ProADM and the CT-proET-1/MR-proADM ratio (0.75 (0.67–0.85) and 0.82 (0.75–0.89); 95% CI respectively). In addition, baseline MR-proADM levels and fluid balance over the five-day period following the admission of ICU were strongly correlated (Rho = 0.41; *p* < 0.001) [20]. 

In a cohort of 128 patients referred to the emergency department with high fever and suspected severe infections such as sepsis, lower respiratory tract infections, urinary tract infections, gastrointestinal infections, soft tissue infections, central nervous system infections, or osteomyelitis, Travaglino et al. found a correlation in the sepsis-septic shock groups between the APACHE II score and MR-proADM [21]. Investigating 120 patients, Valenzuela Sanchez et al. concluded that initial MR-proADM levels helped to identify the infectious origin in patients with systemic inflammatory response syndrome (SIRS) and organ dysfunction [22]. MR-proADM levels and its clearance on day 5 after admission were the most effective biomarkers for determining unfavorable evolution and the risk of mortality in patients with severe sepsis admitted to the ICU [22].

In 2005, Christ-Crain et al.’s observational study of MR-proADM as a prognostic marker in sepsis in 101 patients proposed its use as a new prognostic biomarker in critically ill patients with varying severities of sepsis. The ability of MR-proADM to predict outcomes was similar to that of APACHE II and SAPS II scores [23]. De la Torre-Prados et al. found that MR-proADM showed the best association with 28-day mortality and also had prognostic value (log rank test: *p* = 0.001). Statistical significance was also seen in the Cox regression analysis (*p* < 0.001) for 100 patients with a relative risk 1.26 times higher than baseline for each mmol/L of increase in MR-proADM [24].

In 71 patients with severe sepsis and septic shock, Andaluz-Ojeda et al. also concluded that proADM was the only biomarker to show significant differences between survivors and non-survivors for concentration in plasma at the three time points analyzed (day 1, 3, and 7); it was the biomarker with the best diagnostic accuracy for predicting mortality in the ICU in the AUROC analysis [25]. In 48 septic patients (15 of whom had septic shock), Schuetz et al. found mean MR-proADM levels at admission to be below 1 nmol/L in non-septic critical patients, 2.6 nmol/L in patients with sepsis, and 8 nmol/L (*p* < 0.01) in patients with septic shock [26]. Lundberg et al. also showed that MR-proADM and high sensitivity troponin T (hsTNT) levels were significantly higher among 7-day and 28-day non-survivors [27].

In critically ill children, Rey et al. found that MR-proADM, CT-proET-1, and PCT concentrations above 0.80 nmol/L, 123 pmol/L and 2 ng/mL, respectively, could be used by clinicians to identify those at higher risk of death [28]. Miguel et al. also defined the following reference intervals for potential sepsis markers among newborn infants measured in cord blood plasma with or without risk factors of sepsis: CT-proET-1: 72 pmol/L (39–115); MR-pro-ADM: 0.84 nmol/L (0.5–1.38); and MR-proANP: 163 pmol/L (76–389) [29].

### 3.2. MR-proADM Levels and Sepsis in Burn Patients 

Burns are also associated with increased levels of MR-proADM, but the dynamics regarding sepsis onset after burn injury need to be studied in greater depth. Assessing PCT and MR-proADM as biomarkers of sepsis in 42 burn patients with >15% total body surface area, Gille et al. found that in the patients who developed sepsis (*n* = 27, 64.3%) MR-proADM and PCT levels were significantly higher (*p* < 0.001) on days categorized as septic, with an increase of 31% and at least 0.015 nmol/L (area under curve 0.76) or of >39% and at least 0.15 µg/L (area under the curve 0.83), respectively, for the detection of sepsis. Interestingly, the increase in MR-proADM was recorded one day earlier than in PCT, so the authors concluded that it may be more suitable for use in early recognition of sepsis [30].

### 3.3. MR-proADM Levels in Sepsis with Underlying Malignancies

In malignancies, the origin of fever should be distinguished from infectious and non-infectious causes, such as tumor burden, blood transfusions, or medications. Biomarkers have also been investigated for the diagnosis of infection [31]. Al Shuaibi et al. studied 340 febrile patients (103 with definite sepsis, and 159 with systemic inflammatory response syndrome, SIRS) with hematologic malignancies for the presence of sepsis, SIRS, documented infections, and response to antimicrobial therapy. Only initial MR-proADM levels were found to be significantly higher in patients with localized bacterial infections than in those with no documented infection (*p* = 0.019) and significantly higher in patients with definite sepsis than in those with SIRS (*p* = 0.023) [32].

Debiane et al. also investigated the utility of biomarkers as predictors of sepsis and blood stream infections in 114 febrile critically ill patients with cancer (55 hematologic malignancy, 59 solid tumor). They found that AUROCs for bloodstream infection diagnosis were significantly higher for MR-proADM (0.70; 95% CI, 0.59–0.82) and PCT (0.71; 95% CI, 0.60–0.83) than for CRP (0.53; 95% CI, 0.39–0.66) (*p* = 0.021 and *p* = 0.003, respectively). With regard to patients’ response to antimicrobial therapy, MR-proADM, PCT, and CRP levels all decreased significantly from baseline to follow-up in responders (*p* = 0.002), while only MR-proADM increased significantly in non-responders (*p* < 0.001) [33]. 

## 4. Discussion

In septic patients, early diagnosis and stratification or triage is vitally important for the initiation of appropriate treatment. Our review provides new information on the diagnostic and prognostic role of MR-proADM in sepsis and septic shock.

Statistical analysis could not be performed due to the heterogeneity of the results. In order to define the exact role of MR-proADM as a biomarker for sepsis and septic shock, randomized controlled trials and more collaborative prospective studies should be performed with large numbers of patients.

Due to the complex structure of the septic response, a combination of several tests is likely to be more effective for diagnosis and management than a single ideal biomarker. Angeletti et al. investigated the combined measurement of biomarkers in 104 patients with sepsis and found a better post-test probability with the combination of PCT with MR-proADM; the combination of PCT, MR-proADM, and TNF-α showed the best result in the early diagnosis of sepsis [34]. In both sepsis and localized infections, Angeletti et al. found that the combined use of PCT and MR-proADM increased the post-test probability of diagnosing bacterial infections compared to PCT alone [35].

Although PCT levels are affected by the pathogen (for example, high levels for Gram-negative sepsis), levels of MR-proADM seem to be related to the degree of organ failure and severity of infection rather than the causative agent such as the bacterium or yeast [36]. Angeletti et al. investigated 200 patients with sepsis according to source (Gram-positive and Gram-negative, yeast, and polymicrobial); the AUC for MR-proADM for each were 0.96 (*p* < 0.01), 0.94 (*p* < 0.001), 0.99 (*p* < 0.001), and 0.98 (*p* < 0.001), respectively [37].

MR-proADM levels for viral infections as the source of sepsis and septic shock have not been previously studied. However, Valenzuela-Sanchez et al. reported that MR-proADM plasma levels measured in influenza A pneumonia without bacterial co-infection were more useful than PCT or CRP for predicting the risk of mortality and need for mechanical ventilation [38,39]. In addition, Guignant et al. found significantly elevated plasma MR-proADM concentrations in patients who developed nosocomial infections after a septic shock episode (*p* = 0.043) [40]. Thus, the follow-up of MR-proADM levels may represent a major breakthrough in the monitoring of infectious risk in the ICU patient.

MR-proADM levels are also useful in assessing patients’ evolution. PCT levels may normalize even though the severity persists, and MR-proADM becomes even more useful during evolution, with increased AUROC values in the mortality prognosis by exceeding values of 0.8 in the data shown in several studies. The results also improve along with other biomarkers or severity scores [19,20,22,27,32,33,40].

Our review has some limitations. First of all, the studies present data from high income or developed countries. In low- and middle-income countries, due to lack of resources, diagnosis and triage of sepsis should be performed prior to hospital admission; therefore, physical examination plays an important role [41]. In a study of African countries, Baelani et al. found that 37% did not have the capability to measure lactate [42]. Secondly, the source of sepsis should be considered for the diagnosis and management. The studies published differentiate between the bacterial sources of sepsis and septic shock, but bacteria are not the only causative pathogens: MR-proADM should also be investigated in fungal, viral, and parasitic infections. Although some randomized controlled trials and meta-analyses of the role of MR-proADM in predicting severity and outcome in community-acquired pneumonia patients have been published in order to define the exact role of MR-proADM as a biomarker for sepsis and septic shock, we recommend the performance of randomized controlled trials and more collaborative prospective studies with large numbers of patients specifically in this group of patients [11,43]. Overall, regarding the role of MR-proADM in sepsis and septic shock patients, this review corroborates a prior report which stressed its importance in combination with other biomarkers or organ failure score systems in accordance with the concept of precision medicine [44,45]. A recent systematic review and meta-analysis that assessed both ADM and MR-proADM in patients with sepsis found that the AUC for sROC (summary receiver operating characteristic) was 0.80 (95% CI, 0.77–0.84), which was greater than the results of published meta-analyses of procalcitonin, troponin, and lactate clearance [46]. Finally, the systematic literature review ended in July 2017 and this is an active area of research.

In spite of these limitations, this article has research implications for the future. Perhaps the most interesting value of MR-proADM in sepsis does not lie in its isolated prognostic value (according to the different studies, the molecule presents a modest AUROC that ranges between 0.7 and 0.8 for mortality) or in its diagnostic role (currently poorly demonstrated) but in the combination with other severity scales (such as the risk of early admission to intensive care unit (REA-ICU) score, PSI, CURB-65, SOFA, or APACHE II scores). In this way, many authors have shown how using a combination of the biomarker plus several scales has significantly improved the prognostic value of the first in the field of pneumonia and sepsis. Recent work by Angeletti et al., Andaluz Ojeda et al., and Spoto S et al. demonstrate the interesting potential of the molecule in this aspect [19,34,47]. Finally, identifying drug-response phenotypes is a priority. As emphasized by a recent ESCMID position paper towards precision medicine in sepsis, when searching for biomarkers it is essential to assess their clinical utility [48]. Investigators need to identify biomarkers that are more or less likely to respond to specific interventions (predictive markers).

## 5. Conclusions

MR-proADM has been identified as a prognostic marker that is able to stratify the mortality risk in sepsis patients with different degrees of organ failure. It may be helpful in the early identification and individual risk assessment of sepsis and may also facilitate the subsequent clinical management of sepsis and septic shock. Due to the complex structure of the septic response, it is likely that a combination of several tests may be more effective for diagnosis and management than a single ideal biomarker. MR-proADM levels may also be useful for diagnosis or prognosis of sepsis and septic shock in patients with malignancy or in burn patients.

## Figures and Tables

**Table 1:** Population-Intervention-Comparison-Outcome (PICO) Framework.

Population	Intervention	Comparison	Outcome
**In adults (>18 year old)** Patients with sepsis, severe sepsis, or septic shock	Determining of pro-adrenomedullin/MR-proADM levels	Other biomarkers or score systems	Assessing of diagnostic or prognostic value (via mortality rates) of pro-adrenomedullin/MR-proADM levels
**In children** Patients with sepsis, severe sepsis, or septic shock	Determining of pro-adrenomedullin/MR-proADM levels	Other biomarkers or score systems	Assessing of diagnostic or prognostic value (via mortality rates) of pro-adrenomedullin/MR-proADM levels

PCT: procalcitonin; CRP: C-reactive protein; MR-proADM: mid-regional pro-adrenomedullin; TBSA: total body surface area; MR-proANP: mid-regional pro-anti natriuretic peptide; CT-proET-1: C terminal pro-endothelin 1; PSI: pneumonia severity index; APACHE-II and SOFA (sequential organ failure assessment): score systems.

**Table 2:** Main Characteristics and Administrative Strategy of the Included Studies.

Study and Year	Country	Characteristics	No of Patients	Classification of Patients	Comparison	Results	Sensitivity and Specificity Rates for MR/ProADM with Cut-Off Levels and Area Under Curve (AUC) Levels
**In Adults**							
Schuetz Ph. et al. TRIAGE study, 2015	Switzerland, France, and USA	Multinational, prospective, observational	7132	Consecutive medical patients presenting with a medical urgency	Copeptin, PCT	For the prediction of death, ProADM significantly improved regression models, also improved clinical models for prediction of ICU admission and high initial treatment urgency	(Cut-off: none mentioned but median level of proADM in non-survivors: 1.8 nmol/L) (AUC: 0.83)
Enguix-Armada A. et al., 2016	Spain	Single center, case-control cohort	388	Severe sepsis, septic shock *	CRP, PCT, presepsin, and MR-proADM	PCT, MR-proADM, and presepsin are complementary markers that could be of great help in the management of septic patients when they are measured in the first 24 h after ICU admission	75.8% and 85.9% (Cut-off: 1.11 nmol/L) (AUC: 0.815)
Al Shuaibi M. et al., 2013	USA	Single center, prospective, observational	340	Sepsis, SIRS * with hematologic malignancies	PCT	ProADM had the advantage of predicting localized bacterial infection and differentiating sepsis from SIRS	78% and 35% for bloodstream or localized infections (Cut-off: 0.85 nmol/L) (AUC: 0.601)
David-Andaluz-Ojeda et al., 2017	Spain and France	Two centers, prospective observational cohort	326	Sepsis or septic shock *, **	PCT, CRP, Lactate	MR-proADM accuracy to predict mortality is not affected by the degree of organ failure	83% and 61% for SOFA ≤ 6 (Cut-off: 1.79 nmol/L) (AUC: 0.79)
Angeletti S. et al., 2013	Italy	Single center, case control	320	Sepsis, severe sepsis, SIRS *	PCT	Combined use of PCT and MR-proADM may substantially improve the early diagnosis of sepsis	89% and 96.7% (Cut-off: 1 nmol/L) (AUC: 0.921)
Angeletti S. et al., 2015	Italy	Single center, retrospective	205	Sepsis, severe sepsis, and septic shock *	PCT, IL-6, IL-10, TNF-α, and MCP-1	The combination of PCT with other markers should expedite diagnosis and treatment of sepsis optimizing clinical management	92% and 92% (Cut-off: 1 nmol/L) (AUC: 0.950)
Angeletti S. et al., 2015	Italy	Single center, case control	182	Sepsis, severe sepsis/septic shock *	PCT	PCT and MR-proADM combination improved the diagnosis of bacterial infection and contributed to prognosis and antibiotic therapy effectiveness	85.71% and 97.5% (Cut-off: 1.06 nmol/L) (AUC: 0.929)
Charles PE. et al., 2017	France	Single center, prospective, observational	173	Sepsis, septic Shock *	PCT, copeptin, and CT-pro-endothelin 1 (CT-ProET 1)	MR-ProADM on admission was the best predictor of short-term clinical outcome if compared to others and baseline levels and fluid balance over the 5-day period following ICU admission were strongly correlated	>80% sensitivity rate (Cut-off: 5.19 nmol/L) (AUC: 0.75)
Valenzuela-Sanchez F. et al., 2015	Spain	Single center, prospective, observational	120	Severe sepsis, SIRS *	PCT, CRP	Initial MR-proADM levels helped to identify the infectious origin in patients with SIRS and organ dysfunction. MR-proADM levels and its clearance at the 5th day following admission were the most effective biomarker to determine unfavorable evolution and the risk of mortality in patients with severe sepsis admitted to the ICU	80% and 93.7% (Cut-off: 1.4256 nmol/L) (AUC: 0.947)
Travaglino F. et al., 2012	Italy	Multicentric, observational	128	Severe infections such as sepsis *	PCT, APACHE-II	Supporting the prognostic role of MR-proADM and PCT in that setting, as demonstrated by the correlation with the APACHE II score	(AUC: 0.694) (Cut-off: none mentioned but median value: 0.85 nmol/L in patient group)
Debiane L. et al., 2014	USA	Single center, prospective, cohort	114	Critically ill patients with cancer *	PCT, CRP	In critically ill patients with cancer, pro-adrenomedullin and PCT both had a promising role in predicting bloodstream infections in a manner more helpful than CRP	67% and 68% for blood stream infections (Cut-off: 2.2 nmol/L for blood stream infections) (AUC: 0.70)
Christ-Crain M. et al., 2005	Switzerland	Single center, case-control	101	SIRS, sepsis, severe sepsis, and septic shock *	APACHE-II, SAPS-II, PCT, CRP, IL-6	Prediction outcome is similar to those of the APACHE II and the SAPS II scores; MR-proADM found as a prognostic biomarker in critically ill patients with different severities of sepsis	83.3% and 87.8% (Cut-off: 3.9 nmol/L) (AUC: 0.81)
De La Torre-Prados MV. et al., 2016	Spain	Single center, prospective, observational	100	Septic shock *	PCT, CRP, lactate, APACHE-II, SOFA	MR-proADM levels measured on admission correlated with 28-day mortality in patients with septic shock	79% and 61% (Cut-off: 1.2 ng/mL)
Guignant C. et al., 2012	France	Single center, observational	98	Septic shock *	C-terminal-provasopressin, midregional-proatrial natriuretic peptide, and C-terminal-proendothelin-1	Elevated plasmatic MR-proADM concentration was associated with the development of secondary nosocomial infections after septic shock	(Cut-off: none mentioned but mean value: 0.33 nmol/L) (AUC: 0.86)
Schuetz P. et al., 2007	Switzerland	Single center, observational	95	SIRS, sepsis, and septic shock *	PCT, CT-proET-1, APACHE-II	Endothelin-1 and adrenomedullin precursor peptides gradually increased with increasing severities of infection in critically ill patients.	71.4% and 71.6% (CT-proET-1(pmol/L)/MR-proADM(nmol/L) ratio cut-off: 11) (AUC: 0.76 for MR-proADM)
Andaluz-Ojeda et al., 2015	Spain	Single center, cohort	71	Severe sepsis and septic shock *	PCT, CRP, APACHE-II, SOFA	ProADM was the only biomarker showing significant differences between survivors and non-survivors for concentration in plasma in the three time points (day 1, 3, and 7) analyzed.	(Cut-off: 0.94 nmol/L) (AUC: 0.75)
Lundberg OH. et al., 2016	Sweden	Single center. cohort	53	Septic shock *	MR-proADM, CT-proET-1, hsTNT, and left ventricular systolic functions	Levels of MR-proADM and CT-proET-1 were significantly higher among patients with myocardial injury and were correlated with left ventricular systolic dysfunction. MR-proADM and hsTNT were significantly higher among 7-day and 28-day non-survivors.	80% and 60% (Cut-off: 3.5 nmol/L) (AUC: 0.730)
Suberviola B. et al., 2012	Spain	Single center, prospective, observational	49	Severe sepsis, septic shock due to CAP	PCT, CRP, PSI	MR-proADM levels correlate with increasing severity of illness and death and offer additional risk stratification in high-risk CAP patients	53% and 84% (Cut-off: 4.86 nmol/L) (AUC: 0.72)
Gille J. et al., 2017	Germany	Single center, prospective, observational	42	Sepsis * (TBSA >15% burned patients)	PCT	PCT displayed higher specificity and sensitivity, while MR-proADM may be more suitable for the early recognition of sepsis	63% and 80% (Cut-off: 0.15 nmol/L variance) (AUC: 0.76)
Pereira J. et al., 2016	Portugal	Single center, prospective, cohort	19	Severe CAP*	SAPS-II, SOFA	In severe CAP patients, a decrease in MR-proADM serum levels in the first 48 h after ICU admission was a good predictor of clinical response and better outcome	(Cut-off: none mentioned but with the percent change of MR-proADM in 48 hours, AUC: 0.80 for in hospital mortality)
**In Children**							
Miguel D. et al., 2011	Spain	Single center, case control, prospective observational	267	Newborn infants with or without risk factors of sepsis *	CT-proET-1, MR-proANP, and PCT	The median values (reference interval) of CT-proET-1, MR-pro-ADM, and MR-proANP measured in cord blood plasma were 72 pmol/L (39–115), 0.84 nmol/L (0.5–1.38), and 163 pmol/L (76–389), respectively	Reference range for MR-proADM: 0.55 to 1.38 nmol/L
Rey C. et al., 2013	Spain	Two centers, prospective observational	254	Critically ill children *	CT-proET-1, PCT, and CRP	In critically ill children, high levels of MR-proADM, CT-proET-1, and PCT were associated with increased prediction of mortality risk scores	93% and 76% (Cut-off: 0.79 nmol/L) (AUC: 0.87)
**Opinion Paper**							
Di Somma S. et al., 2013	Italy	Opinion paper	-	Sepsis	PCT, inducible protein 10 (IP10), Group IV phospholipase A2 type II (PLA2 II), neutrophil gelatinase-associated lipocalin (NGAL), natriuretic peptides, mature adrenomedullin (ADM), copeptin, thrombopoietin, Mer receptor, and red blood cell distribution width (RDW)	Biomarkers including MR-proADM can represent an appealing perspective in the diagnosis and management of patients with sepsis. Nevertheless, at the moment, it is not still clear if it is better to use a multimarkers approach or if a single, most appropriate, biomarker exists	
**Review**							
Valenzuela Sanchez F. et al., 2016	Spain	Review	-	Sepsis, septic shock	CRP, PCT	The MR-proADM levels are more effective than procalcitonin (PCT) and C-reactive protein (CRP) levels to determine an unfavorable outcome and the risk of mortality in patients with sepsis admitted to the ICU. It has also proved useful in patients diagnosed with organ dysfunction of infectious etiology	

ICU: intensive care unit; SIRS: systemic inflammatory response syndrome; IL: interleukin; TNF: tumor necrosis factor; MCP: monocyte chemoattractant protein; SAPS: simplified acute physiology score; hsTNT: high sensitivity troponin T; CAP: community acquired pneumonia; PCT: procalcitonin; CRP: C-reactive protein; MR-proADM: mid-regional pro-adrenomedullin; TBSA: total body surface area; PSI: pneumonia severity index; APACHE-II and SOFA: score systems; AUC: area under the receiver operating characteristic curve. * American College of Chest Physicians/Society of Critical Care Medicine Consensus Conference and Surviving Sepsis Campaign Criteria. ** SEPSIS-3 Criteria.
